# Chain formation mediated by *Escherichia coli* immunoglobulin-binding proteins EibD and EibG depends on expression levels and localization of proteins

**DOI:** 10.1038/s41598-025-22967-3

**Published:** 2025-10-09

**Authors:** Lena-Sophie Swiatek, Katharina Schaufler, Jack C. Leo

**Affiliations:** 1 Department of Epidemiology and Ecology of Antimicrobial Resistance, Helmholtz Institute for One Health, Helmholtz Centre for Infection Research HZI, Greifswald, Germany; 2 University Medicine Greifswald, Greifswald, Germany; 3 Antimicrobial Resistance, Omics and Microbiota Group, Centre for Systems Health and Integrated Metabolic Research, Department of Biosciences, Nottingham Trent University, Nottingham, UK

**Keywords:** Adhesion, Autoaggregation, Cellular localization, Chain formation, *E. coli* immunoglobulin-binding protein, Trimeric autotransporter adhesins, Proteins, Fluorescence imaging, Genetic engineering, Microbiology techniques, Microscopy, Confocal microscopy, Phase-contrast microscopy, Software, Molecular modelling, Mutation, Prokaryote, Bacterial pathogenesis, Bacterial structural biology, Biofilms, Pathogens, Molecular modelling, Biochemistry, Microbiology, Molecular biology, Structural biology

## Abstract

*Escherichia coli* expresses immunoglobulin-binding proteins (Eib), a subgroup of trimeric autotransporter adhesins (TAA). Subtypes of Eib proteins mediate unique chain-like adherence patterns and autoaggregation. This study investigates the mechanisms underlying chain formation by EibG and EibD; a chain-forming phenotype of the latter has not been previously described. Using constitutive expression systems, we demonstrate that moderate-level expression of EibG and EibD lead primarily to chain formation, whereas higher expression levels predominantly result in clump formation. Notably, chain and clump formation are not mutually exclusive and can occur simultaneously. Selective deletion of the full head domain, but not the N-terminal domain alone, abolished chain formation, highlighting its critical role. Fluorescence microscopy of mixed cultures showed that chains form through homotypic protein-protein interactions. Investigation revealed EibD and EibG were predominantly localized at cell poles, corresponding to sites of intercellular contact. Functional investigations showed that chain-forming strains exhibited enhanced adhesion to plastic surfaces, a key step in biofilm formation, without affecting autoaggregation. These showed Eib-mediated chain formation depends on protein expression levels, domain architecture, and localization, contributing to bacterial adhesion and potentially pathogenicity. Understanding interactions provides insights into TAA-mediated chain formation and autoaggregation.

**Supplementary Information:**

The online version contains supplementary material available at 10.1038/s41598-025-22967-3.

## Introduction

The species *Escherichia coli*, comprising both commensal and pathogenic representatives, is well studied. However, especially multi-drug resistant isolates are a major public health threat^1^. It is a versatile organism and pathogen reflected by a multitude of proteins presented on its cell surface^2^. This includes adhesins, pili, and fimbriae, which mediate attachment to host tissues and surfaces and play a critical role in colonization and infection^3,4^. Once adhered, *E. coli* can form biofilms that enhance resilience to environmental stressors, immune responses, and antibiotics^5^. By that, they contribute to the persistence and pathogenicity of *E. coli* in host organisms and various environments^3,5^.

Different highly specialized protein secretion systems exist in Gram-negative bacteria that ensure protein transport across the inner and outer membranes^6^. Proteins belonging to the type 5 secretion system utilize the Sec translocation machinery for crossing the inner membrane^7^. These proteins are characterized by a β-barrel domain that inserts into the outer membrane^8,9^. No cytosolic energy sources for translocation across the outer membrane is needed resulting in the term autotransporter^10,11^. Trimeric autotransporter adhesins (TAAs, Type 5c secretion systems) are a group of obligate homotrimeric proteins that mediate their own transport across the outer membrane, a process that involves the β-barrel assembly machinery but remains poorly understood^11–14^. These proteins are recognized as key virulence factors in Gram-negative bacteria, contributing to diverse functions, including immune evasion, serum resistance, host cell adhesion and invasion, and biofilm formation^15^. TAAs consist of a N-terminal signal peptide enabling Sec-dependent inner membrane translocation and their outer membrane translocation pore is formed by conserved 12-stranded β-barrel domains with each protomer contributing four of the stands^8,16–18^. The extracellular region or passenger contains one or more right- and/or left-handed coiled-coiled regions, as well as globular domains forming ‘heads’. Some TAAs, such as the prototypical *Yersinia* adhesin YadA, have a single head atop a coiled-coil stalk and thus resemble a lollipop^19^. However, other TAAs are more complex, with a number of heads interspersed with stretches of coiled coil^20^. In addition, smaller minidomians such as the saddle or an insertion of a 3-stranded β-meander into a coiled-coil segment (FGG motifs) may be present^21,22^.

One subgroup of TAAs comprises the immunoglobulin (Ig)-binding proteins (Eib) found in *E. coli*, including several distinct proteins^23,24^. These proteins mediate non-immune binding to IgG and IgA Fc regions^25^. They are prevalent in Shiga toxin-producing *E. coli* (STEC) but are also found in commensal strains, suggesting a potential role in intestinal adhesion^26^. Among these, EibG has been described as mediating a unique chain-like adherence pattern (CLAP) on both human and bovine cells^27^. Static growth, microaerophilic conditions, and alkaline pH induce EibG expression in STEC while agitation and decreased temperature is repressive^28^. These observations indicate that chain formation by EibG is not host-specific and likely represents a bacteria-to-bacteria adhesion mechanism that also occurs in liquid culture^26,27^.

Eib proteins are known to induce autoaggregation, which manifests as clump formation under specific conditions^21,29^. Similarly to other multifunctional proteins like YadA, which binds to collagen and other extracellular matrix components and mediates serum resistance, EibG may have additional roles beyond adhesion. Despite its significance, little is known about the underlying mechanism by which EibG facilitates chain formation. Here, we investigated how EibG mediates chain formation. Our results demonstrate that EibG and also EibD can mediate chain formation under certain conditions. Chain formation depends on homotypic interactions of Eib proteins enriched at the cell contact sites and also on the expression levels of the protein. Understanding this mechanism in detail could provide insights into the broader biological functions of TAAs and their role in bacterial adhesion.

## Results

### Expression levels influence chain formation

**Fig. 1:**
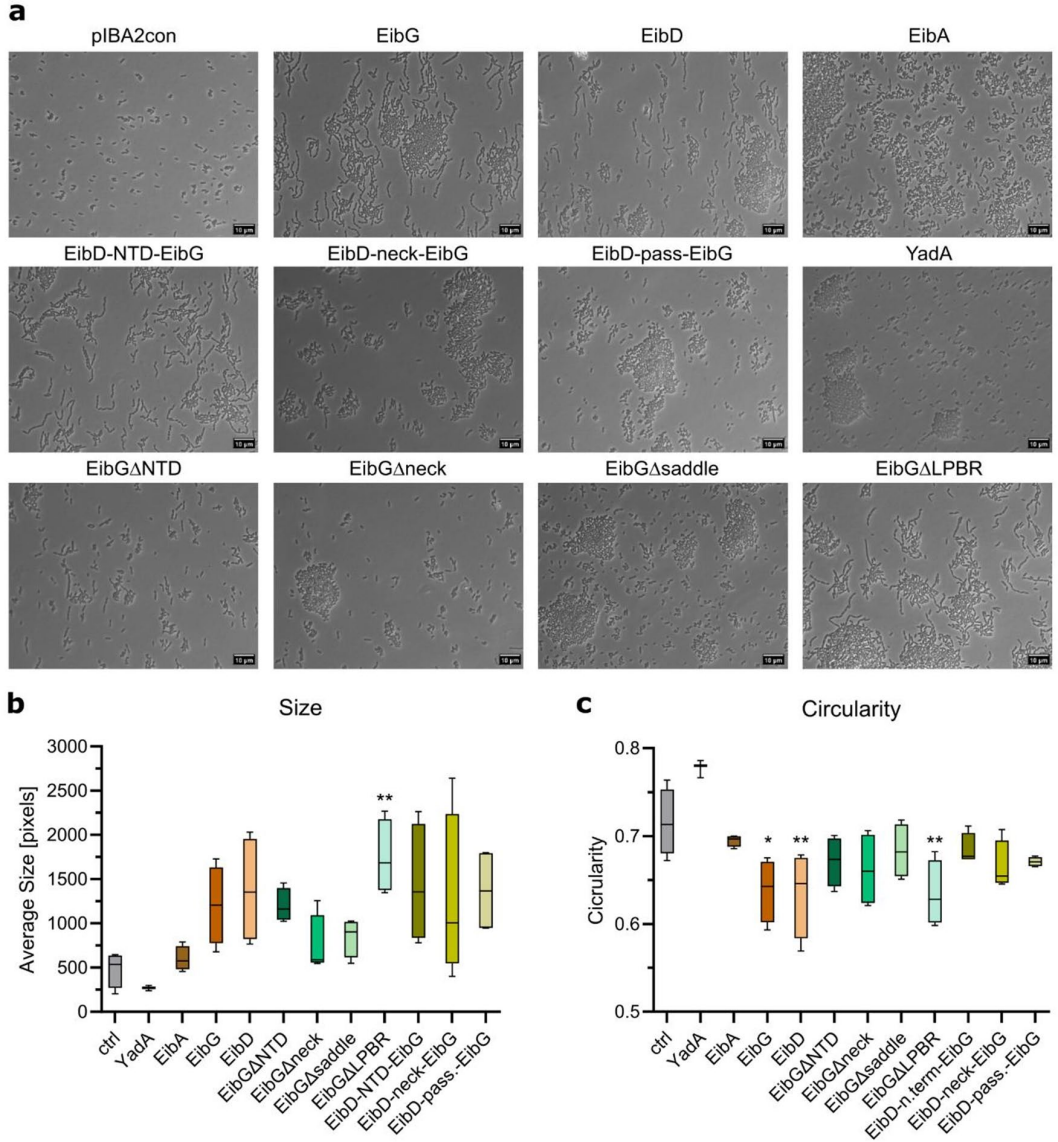
Evaluation of chain vs. clump formation. (**a**) Bright field microcopy was performed after incubation of the indicated cultures to differentiate between individual cells, clumps and chains. Cells were taken from the bottom of the tube transferred onto a prepared objective slide after gentle resuspension. The scale bar in the bottom right corner indicates 10 μm. pIBA2con is the empty vector. For an explanation of the various deletion constructs, refer to Fig. 2b. (**b** + **c**) Ten semi-randomly selected pictures were evaluated to obtain their size and circularity using Fiji (ImageJ) and the average was calculated. This was performed for four biological replicates each. Boxplots show the median, interquartile range (25th to 75th percentile), and whiskers representing the minimum and maximum values. One-way ANOVA with Dunnett’s post-hoc test was used for multiple comparisons to the control group (*p*-value: * 0.01–0.05; ** 0.001–0.01).

While auto-aggregation and clump formation are known phenomena mediated by Eib proteins^21,29^, previous studies indicated a CLAP for cells expressing EibG while adhering to human cells^27^. However, we found that Eib proteins, including EibG, formed clumps when expressed at high levels using a strong inducible promoter (Supplementary Fig. 1 and Supplementary Fig. 2). It is known that the P_*ara*BAD_ promoter and other standard inducible promoters have an all-or-nothing effect at the cellular level, meaning that any reduction in expression at a population level is due to fewer cells being induced, but induced cells continue to produce high levels of the cloned protein^30–32^. Therefore, to evaluate the influence of lower expression levels on the chain formation, we cloned different *eib* variants into a plasmid with a medium-low strength promotor for constitutive expression and transformed *E. coli* TOP10 cells with respective constructs. Using this system, we were able to show that chain formation is observed when cells express EibG and EibD at low levels (Fig. 1 and Supplementary Fig. 2). By contrast, EibA (with an EibD signal peptide) expression in this system did not lead to chain formation. For EibD and EibG, both clump and chain formation were often observed simultaneously and were not mutually exclusive. Image analysis demonstrated that chain formation is reflected in an increase of particle size but decrease in circularity compared to control cells containing the empty vector and EibA expressing cells, which form small clumps. Cells expressing YadA, the well-studied *Yersinia* ssp. autotransporter protein^23^, only showed clump formation at the analyzed conditions.

### Deletion of head-domain but not N-terminal or left-handed parallel β-roll domain alone depletes the chain forming phenotype

**Fig. 2:**
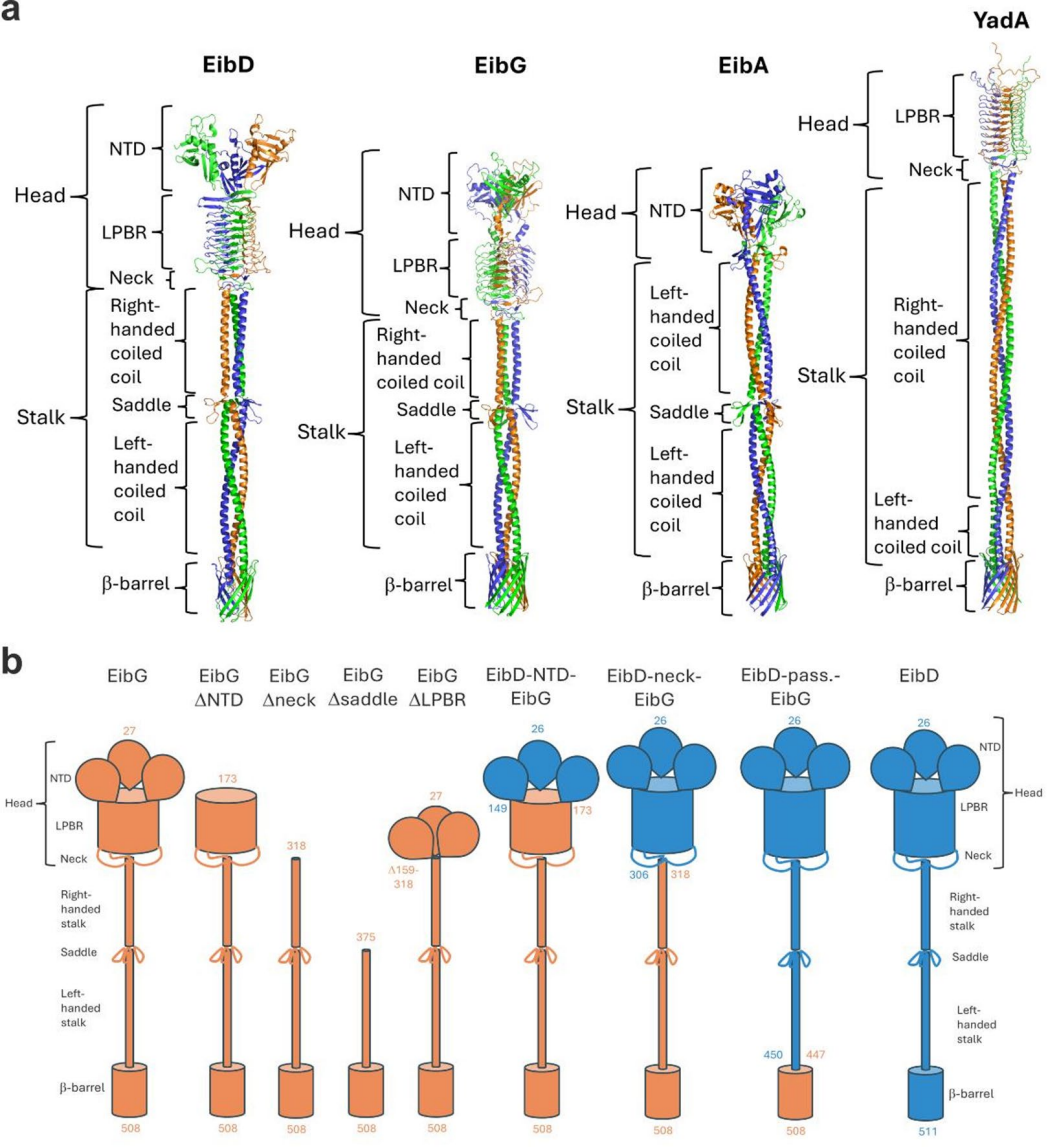
AlphaFold2 models of TAAs and schematic presentation of TAA constructs used in this study. (**a**) The TAAs all display a lollipop-like architecture, with a C-terminal β-barrel, a stalk and a head region. Structural features are indicated. NTD = N-terminal domain, LPBR = left-handed parallel β-roll domain. The three chains of the trimers are colored differently. The figure was produced using PyMOL (Schroedinger). (**b**) Schematic presentation of EibG (orange) and EibD (blue) mature proteins are shown, with the N- and C-terminal residues of each construct highlighted. For chimeric constructs, the start and end residues for each portion of the protein are given in the color corresponding to the protein (orange for EibG, blue for EibG).

A crystal structure encompassing the YadA-like left-handed parallel β-roll domain (LPBR) and the LPBR and coiled coil stalk of EibD has been previously solved^21,33^. However, no structural information has been available for the N-terminal domain (NTD) of this protein. Therefore, we used AlphaFold2^34,35^ to model the full trimers of EibD and EibG, as well as EibA and YadA (Fig. 2). The Eib proteins share highly similar structures at the C-terminus, which includes the β-barrel membrane anchor, a left-handed coiled-coil region and the saddle minidomain. In EibD and EibG, the N-terminal half of the stalk is a right-handed coiled coil, whereas in EibA, this is left-handed. EibD and EibG share a similar bipartite head region encompassing the NTD with a novel fold and the LBPR. EibA lacks a LPBR, having only the NTD. The NTD of these three proteins has a triangular core with two α-helices packing against an antiparallel β-sheet (Supplementary Fig. 3a). In addition, a β-hairpin packs against the longer α-helix to make the ‘tip’ of the upside-down triangle. The NTDs of the three Eib proteins have some differences, particularly in the way they connect to the LPBR domain, or stalk in the case of EibA (Supplementary Fig. 3a). The NTDs have limited structural similarity to the plectin repeats of periplakin, a eukaryotic cytoskeletal linker protein^36^.

EibG and EibD are relatively similar in the region of the LPBR domain, but EibA lacks this domain. We therefore evaluated chain formation on cells constructively expressing EibG with deletion of the LPBR. This revealed that the deletion of the EibG LPBR domain does not prevent chain formation (Fig. 1). Although EibG and EibD both have the potential of chain formation, chain formation for EibG was more pronounced with longer chains.

To identify domains that influence chain formation we introduced domain deletions in EibG and domain swaps between EibG and EibD proteins and validated protein production and trimerisation in the outer membranes (Supplementary Fig. 2). This revealed that deletion or alteration of the NTD does not inhibit chain formation. However, when deleting the head domain, which comprises the NTD, the LPBR, and neck domain, the bacteria formed clumps or remained as individual cells but no chains were observed. The same applied for the deletion of the whole passenger After quantifying the microscopy images, this was reflected in decreased size of the particles with slight increase in circularity. Similar observations were made when exchanging the domains between EibG and EibD. Clump formation resulted in a high variability in particle size and a relatively constant circularity (Fig. 1).

### Chain forming phenotype is induced by homotypic protein-protein interaction predominantly at the cell poles

**Fig. 3:**
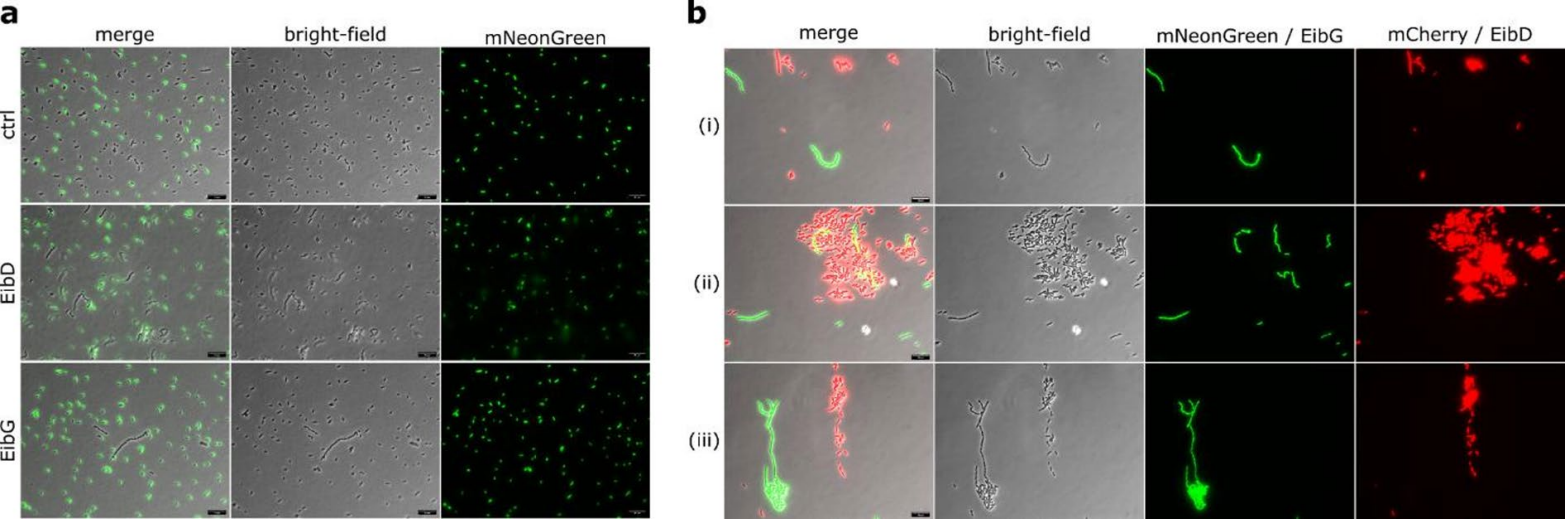
Assessing mixed chain formation. Mixed cultures were set up containing (**a**) mNeonGreen expressing TOP cells and either, pIBA2 (ctrl), EibD, or EibG (**b**) Cells expressing both mNeonGreen and EibG or mCherry and EibD. Combination of bright field and fluorescence microscopy was used to analyze mixed chain formation. The scale bar in the bottom right corner indicates 10 μm.

We were interested in whether interaction of the Eib proteins with themselves or with other surface structures leads to chain formation. Therefore, we set up mixed cultures with an *E. coli* reporter strain expressing mNeonGreen but no TAA and the Eib-expressing strains and performed microscopy (Fig. 3a). In the mixed culture containing the empty vector control, individual cells were observed only. In contrast, chain formation was again detected in the EibG and EibD cultures. These chains consisted exclusively of cells lacking the mNeonGreen signal, while individual mNeonGreen-positive cells were found dispersed around them.

In the next step, we evaluated whether heterotypic chains or clumps formed in a mixed culture of cells expressing mNeonGreen and EibG or mCherry and EibD, respectively. Upon fluorescence-microscopic evaluation, it became evident that chains are exclusively formed through homotypic interactions, as non-TAA expressing cells did not participate in chain formation (Fig. 3b). EibD and EibG-expressing cells did not appear to interact, confirming that the interactions are homotypic and specific to each TAA. However, the additional expression of the mCherry reporter in EibD-expressing cells enhanced the clump-forming phenotype. Sporadically, EibG chains were found within the EibD clumps. However, due to the lack of consistent co-localization and the absence of EibD- and EibG-expressing cells within the same chain, we interpret this observation as likely resulting from nonspecific or incidental interactions rather than a biologically meaningful association.

**Fig. 4:**
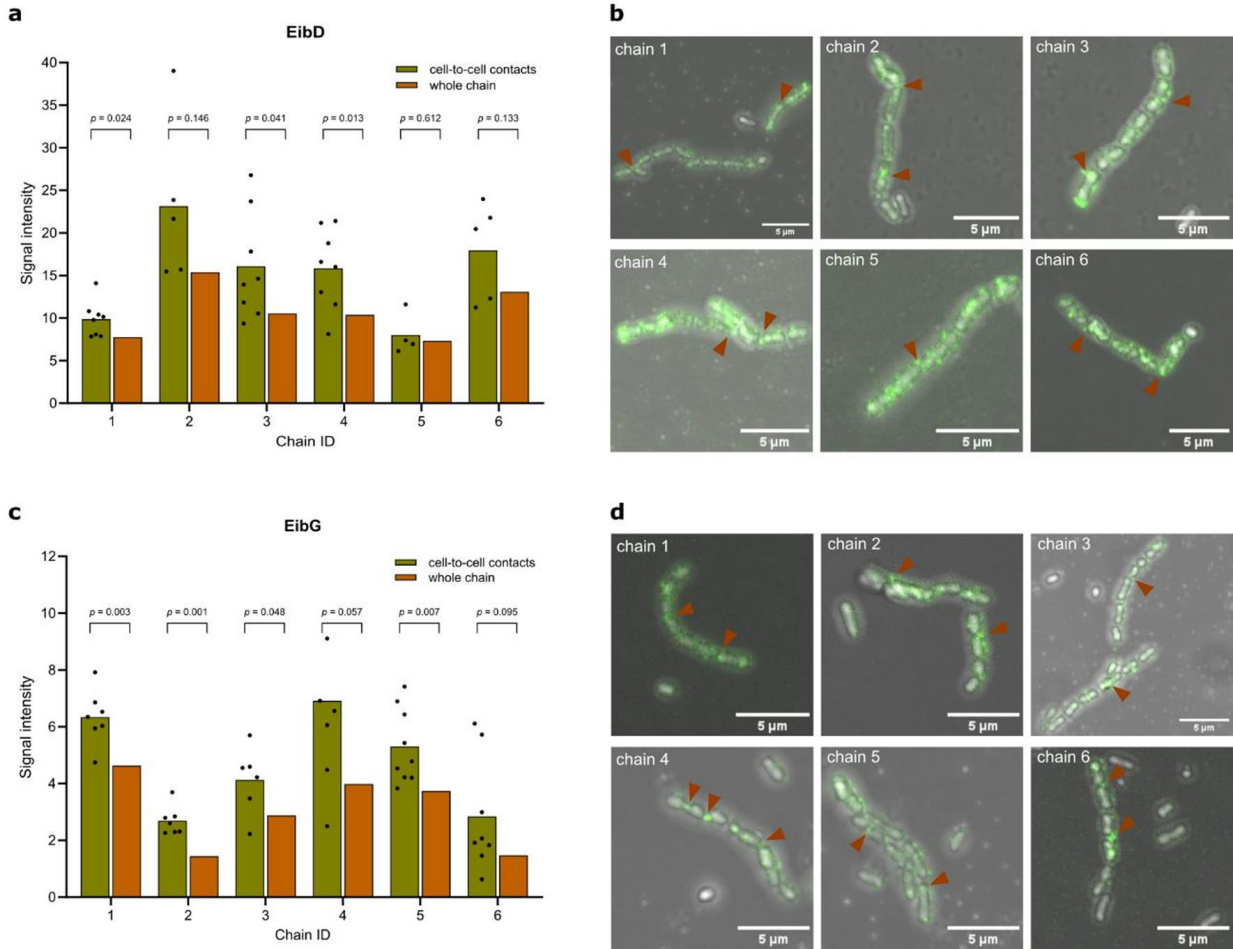
Cellular localization of Eib proteins. (**a** + **b**) EibD and (**c** + **d**) EibG proteins, each with an N-terminal SpyTag, were expressed in E. coli TOP10 cells and labeled with previously purified SpyCatcher protein. (**a** + **c**) Localization of the proteins was assessed by quantifying the fluorescence signal at cell-to-cell contacts compared to the signal along the entire bacterial chain. Significant differences were evaluated by performing a paired t-test (*p-*values are indicated). Representative microscopic images of quantified chains are shown for (**b**) EibD- and (**d**) EibG-expressing cells, with cell-to-cell contacts exhibiting pronounced fluorescence indicated by arrows. The scale bar in the bottom right corner indicates 5 μm.

Moreover, we were interested in whether the chain-forming phenotype is induced by localization of the respective Eib proteins at cell-to-cell contacts. For that, we used EibD and EibG variants with a SpyTag^37^ located at the N-terminus upon cleavage of the signal peptide. Detection was ensured by incubation with a previously purified SpyCatcher-superfolder green fluorescence protein (sfGFP) protein which forms a covalent bond with the SpyTag^38^. Evaluation of the fluorescent signal from confocal fluorescence microscopy revealed that EibD and EibG proteins could be found around the whole cell. However, increased signals were detected at the contact points of the individual cells within a chain, i.e. at cell poles (Fig. 4). Quantification of these signals revealed an increased signal intensity for the cell-to-cell contacts compared to the whole chains, being significantly higher for the majority of chains.

We also investigated the localization in clumps. The high cellular densities within clumps make precise detection and quantification difficult. However, no distinct localization was observed. Both Eib variants were detected around the cells in clumps with some individual cells showing enhanced signals, most likely due to variable accessibility of the fluorescent label within the clumps (Supplementary Fig. 4).

In order to check for specificity of the signal, the Eib-SpyTag variants were incubated with a SpyCatcher-EQ-sfGFP variant, which prevents formation of a covalent bound with the SpyTag. SpyCatcher-EQ-sfGFP resulted in diffuse, low-intensity non-specific signals (Supplementary Fig. 5).

**Fig. 5:**
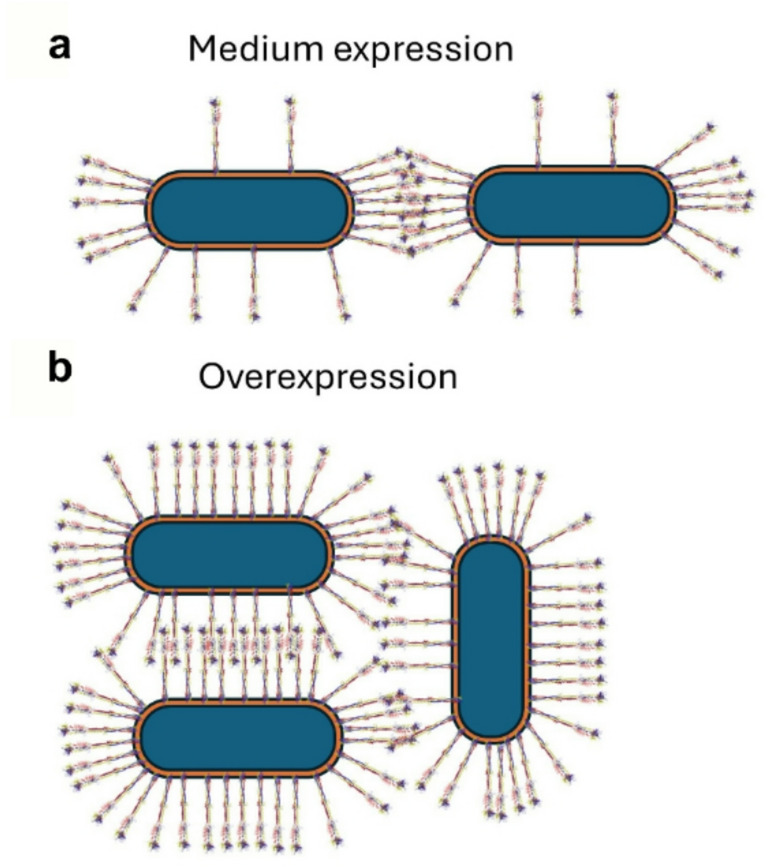
Model for Eib-mediated chain formation. (**a**) At medium expression levels, most protein is located at the bacterial poles, with some spillover onto the lateral sides. However, the low density of adhesins and presumably low individual affinities between Eib molecules means that cooperative binding happens mainly at cell poles, where the density of adhesins is high enough to allow stable interactions. (**b**) At high expression levels, Eibs cover the entire cell at high densities, leading to clump formation as stable interactions can happen at all sides of the cell.

Based on these findings, we propose the following model for chain formation in Eib-expressing cells (Fig. 5): at low expression levels, proteins are preferentially exposed at the cell poles. The high density at the poles leads to homotypic interactions that mediate chain formation. Some spillover from the poles may lead to low levels of protein along the lateral side of the cell, but because of the presumed low affinity of the homotypic interactions, the low protein density here would not support cooperative binding and consequently cells only attach at the poles. At higher expression levels the proteins are more evenly distributed and cooperative interactions can take place at the lateral sides as well, which leads to clump formation. This is indeed what we observe in our current setup, where chain formation and clump formation coexist. We do see some fluorescent signal on the lateral sides of cells in chains, but an enrichment at cell-cell contact sites, which is consistent with this model. Future work will aim to uncover how Eibs are preferentially targeted to the cell poles.

### Chain formation does not impact auto-aggregation but adhesion to polystyrene

**Fig. 6:**
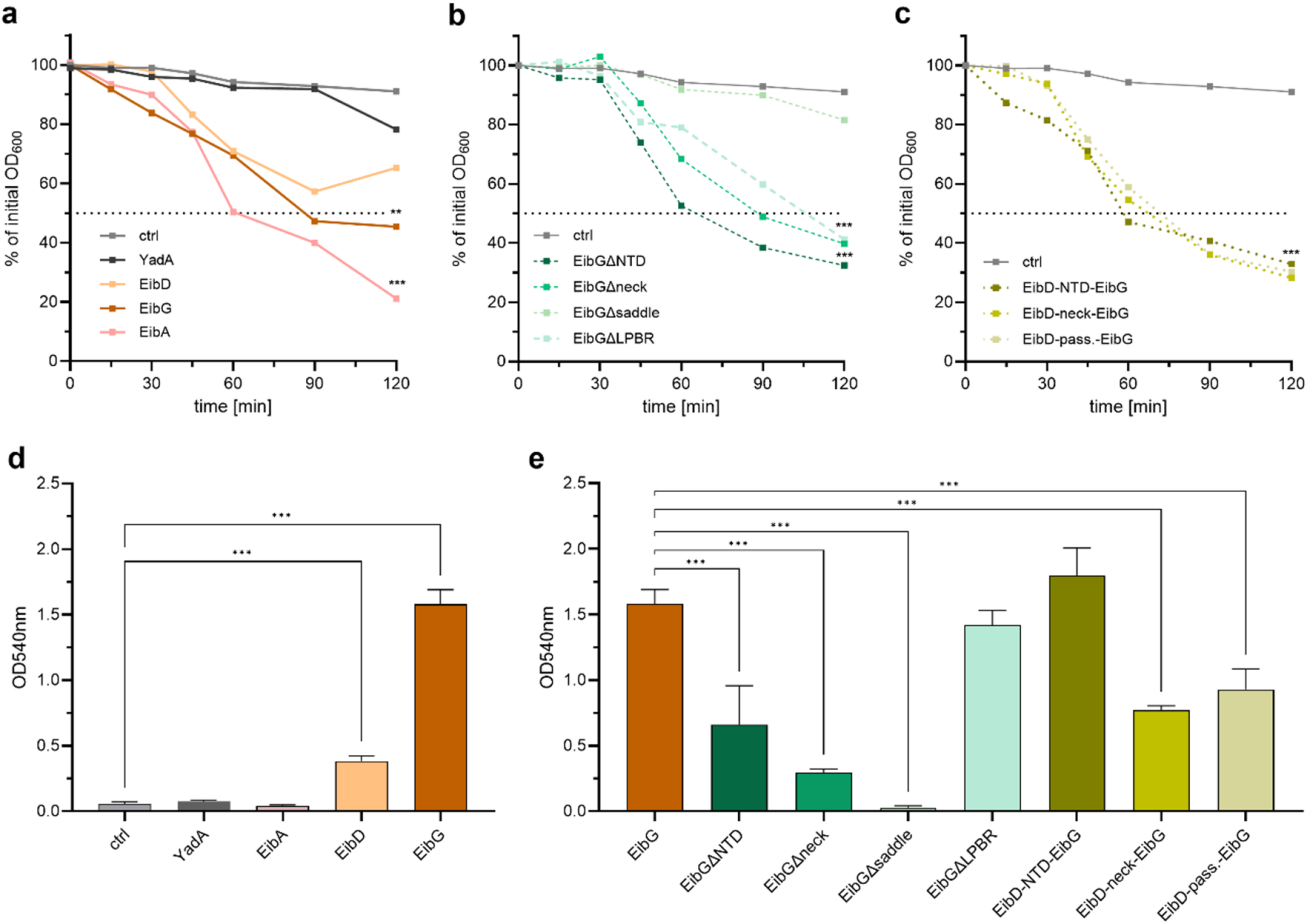
Influence of chain formation on auto-aggregation and biofilm formation. For analysis of auto-aggregation strains were cultivated overnight, mixed by inverting, and then transferred to long glass tubes. The OD_600_ from the top of the cultures was measured regularly in three biological replicates and the mean value and standard deviations are plotted: (**a**) EibD, EibG, and EibA, and YadA expressing wild type cells, (**b**) deletion mutants, (**c**) exchange mutants. (**d** + **e**) Crystal violet assay for the different Eib expressing cells: The cells were seeded into a 96-well microtiter plate were stained with crystal violet staining. Upon solubilization of the stain the OD was measured at 540 nm. The mean value and standard deviation is shown of three biological replicates. One-way ANOVA with Dunnett’s post-hoc test was used for multiple comparisons to the control group (**a**,** b**,** c**, and **d**) or EibG (**e**).

TAAs are known to promote auto-aggregation among other virulence-associated phenotypes like biofilm formation^29^. We tested auto-aggregation of chain-forming bacteria using sedimentation assays (Fig. 6). All wild-type strains demonstrated auto-aggregative phenotypes in contrast to the empty-vector control. None of the domain deletions and domain exchange mutants showed altered auto-aggregation behavior, except for the deletion of almost the entire passenger (EibG∆saddle).

In contrast to auto-aggregation, an alteration in chain formation severely affected the adhesive phenotype on plastic surfaces, which is recognized as the crucial initial step of biofilm formation. For all strains that did not show chain formation, namely cells expressing, EibA EibG∆neck, and EibG∆saddle, the intensity of crystal violet staining was equal to the control or significantly reduced compared to EibG wild-type expressing strains. This was also true for cells with low-level YadA expression, although higher levels of YadA have been shown to promote biofilm formation^29^. For EibA, weak biofilm formation has been demonstrated before^29^. By contrast, strains with the chain-forming phenotype showed increased adhesion. For the NTD deletion mutant a slight decrease in adhesion was observed consistent with the reduction in chain sizes. When comparing the mutants with domain swaps between EibD and EibG, the highest levels of adhesion became evident for the chain-forming EibG variant with the EibD N-terminal domain (EibD-NTD-EibG). When additionally exchanging the head and neck domains or the whole passenger a decrease in staining intensity was observed for the clump-forming mutants compared to the wild-type and the chain-forming mutant (EibD-NTD-EibG).

For testing whether the altered adhesive phenotypes result in different biofilm morphology, we assed macrocolony formation (see Supplementary methods 3). Interestingly, while there was a significant difference in adhesive phenotypes between the chain-forming and chain-deficient strains, no notable variation was observed among the long-term macrocolonies of the EibG wild type and its respective mutant strains. Notably, distinct biofilm phenotypes were observed among the different Eib wild type variants (Supplementary Fig. 6).

## Discussion

This study highlights the critical influence of expression levels, and structural domains on the aggregation behavior of Eib proteins in *E. coli*. The findings offer new insights into the mechanisms driving chain formation and adhesive phenotypes, while also suggesting the predominant localization of EibD and EibG at cell-cell contacts, particularly at the cell poles, contribute to these processes.

The results demonstrated that lower expression levels of EibG and EibD promote chain formation, a phenotype absent in EibA. Note that despite clear chain formation clumps were also still observed for both. Under laboratory conditions, Eib proteins were highly stable, while protein levels differed significantly among different strains^39^. Previous studies described a CLAP for EibG and EibF-expressing strains when adhering to human or bovine cells only while a *eibABCDE*-postive strain does not show a CLAP^27^. This could be explained by the presence of EibA or higher expression levels in general leading to clumping that masks any potential CLAP. Here we demonstrated that chain formation is not only induced by EibG and EibF but also EibD when expressed at moderate levels. The chain formation phenomenon does not require adhesion to cells but occurs in liquid cultures too. The absence of chain formation by EibA suggests that while these proteins share common features, functional differences arise due to sequence and structural variations.

Differences became particularly evident in the LPBR. Although EibA lacks the LPBR region, deletion of this domain in EibG and EibD did not inhibit chain formation. Deletion of the LPBR affected neither adhesion to plastic nor autoaggregation. These findings do not align with earlier reports that the LPBR region of TAAs facilitates homotypic interactions critical for aggregation^40^. Similarly, deletion or exchange of the NTD did not prevent chain formation, resulting in similar adhesion capacities as seen in wild-type EibG expressing cells. By contrast, deletion of the head domain, consisting of the NTD, the LPBR and the neck domain, resulted in disrupted chain formation, and significantly impaired adhesion. Different *eibG* allelic variants exist that show phenotypes differing in length and sequence variation within the LBPR and neck domains^26^. Together, these observations suggest that the domains of the EibG head contribute to autoaggregation and chain formation, so that only when the entire head is deleted are these processes affected.

We observed that chain formation, rather than clump formation, is a key determinant of adhesion in these systems, while biofilm macrocolony morphologies did not differ among the EibG wild type and its mutants. Adhesion is the initial step in host colonization, which in turn is a prerequisite for causing severe pathologies^41^. By shedding light on the molecular determinants of chain formation, a growing body of knowledge may inform the development of strategies to prevent adhesion of pathogenic bacteria as demonstrated for FimH^42,43^. In line with previous reports, no chain or biofilm formation was observed for EibA, which has instead been implicated in immunoglobulin binding and serum resistance^44^. By contrast, autoaggregation is not affected by alteration of the chain-forming phenotype and occurs independently of microscopic clump or chain formation.

We demonstrated that chain formation exclusively occurs between cells expressing EibG or EibD and not among a mixed population of cells expressing no or different Eib variants. Moreover, we hypothesize that the localization at the cell poles is essential for the chain forming phenotype. Polar localization of autotransporters is a known process in *E. coli* that has been extensively studied, e.g. for the type 5a autotransporters IcsA and Ag43 but applies to other membrane proteins as well^45–48^. These studies suggested that these outer membrane proteins are targeted to the pole in the cytoplasm before secretion through first aggregating at the cell pole^49,50^. Subsequent disaggregation allows direct transport through the inner membrane and periplasm to the outer membrane at the cell pole. Multiple factors such as protein sequence determinants, lipopolysaccharide integrity, expression levels and the activity of key proteins, e.g. YidC, DnaK or FtsQ have been suggested to be involved in this process^45,48–51^. However, many of these localization studies were performed in the absence of signal peptides, so the validity of this aggregation/disaggregation model is not fully confirmed. Signal peptides enable Sec-dependent translocation of TAAs into the periplasm^12^. Omitting the signal peptide could easily lead to the formation of intracellular inclusion bodies, which may or may not be representative of the native process when a signal peptide is included.

The results of this study indicate that low-level expression of Eib proteins leads to their initial predominant localization at the cell poles, which later serve as contact points for the initiation of chain formation. The mechanism behind this still requires further investigation. Furthermore, we identified that complex structural features of the Eib proteins are required as prerequisites for mediating the type of intercellular interaction that enables chain formation. The interplay between structural domains and expression levels in regulating bacterial adhesion and biofilm formation has important implications for understanding the pathogenic potential of STEC and related strains.

## Methods

### Bacterial strains and cultivation

The bacterial strains used in the study are listed in Table 1. *E. coli* TOP10 cells were used as host cells for constitute expression of all protein variants with the pASK-IBA2con backbone and a chloramphenicol resistance cassette (Supplementary Fig. 1). High-level expression using the pBAD (Invitrogen) background was induced with 0.2% l-arabinose in either TOP10 or BL21ΔABCF^52^. Bacterial strains were cultivated in LB-Lennox medium or auto-induction medium (AIM) containing 25 µg/mL chloramphenicol, 15 µg/mL tetracycline or 100 µg/mL ampicillin at 37 °C. For cloning and protein expression, strains were cultivated shaking at 150 rpm. Whenever chain formation was induced, shaking was reduced to 30–50 rpm.

**Table 1:** Bacterial strains.

Strain name	Host	Plasmid	Description	Reference
Ctrl	*E. coli* TOP10	pASK-IBA2con	Control strain	Invitrogen
EibA	*E. coli* TOP10	pASK-IBA2con_*eibA*	Constitutive expression of EibA	This study
EibD	*E. coli* TOP10	pASK-IBA2con_*eibD*	Constitutive expression of EibD	This study
EibG	*E. coli* TOP10	pASK-IBA2con_*eibG*	Constitutive expression of EibG	This study
EibG∆NTD	*E. coli* TOP10	pASK-IBA2con_*eibG*∆n.term	Constitutive expression of EibG with deleted N- terminal domain (NTD) [∆Q28-L173]	This study
EibG∆neck	*E. coli* TOP10	pASK-IBA2con_*eibG*∆neck	Constitutive expression of EibG with deletion up to and including the neck domain [∆Q28-V318]	This study
EibG∆saddle	*E. coli* TOP10	pASK-IBA2con_*eibG*∆saddle	Constitutive expression of EibG with deletion up to and including the saddle domain [∆Q28-A375]	This study
EibG∆LPBR	*E. coli* TOP10	pASK-IBA2con_*eibG*∆LPBR	Constitutive expression of EibG with deleted LPBR domain [∆S159-V318]	This study
EibD-NTD-EibG	*E. coli* TOP10	pASK-IBA2con_*eibD*- n.term_*eibG*	Constitutive expression of EibG variant encoding the EibD N-terminal domain EibG[ΔQ28–L173] :: EibD[Q27–E149]	This study
EibD-neck-EibG	*E. coli* TOP10	pASK-IBA2con_*eibD*- neck_*eibG*	Constitutive expression of EibG variant encoding the EibD neck domain EibG[ΔQ28–V318] :: EibD[Q27–V306]	This study
EibD-pass-EibG	*E. coli* TOP10	pASK-IBA2con_*eibD*- pass_*eibG*	Constitutive expression of EibG variant encoding the EibD passenger EibG[ΔQ28–V447] :: EibD[Q27–V450]	This study
YadA	*E. coli* TOP10	pASK-IBA2con_*yadA*	Constitutive expression of YadA	This study
Ctrl-GFP	*E. coli* TOP10	pACYC184-mNeonGreen	Assessing mixed chain formation	This study
EibG-GFP	*E. coli* TOP10	pACYC184-mNeonGreen	Assessing mixed chain formation	This study
EibD-mCherry	*E. coli* TOP10	pACYC184-mCherry	Assessing mixed chain formation	This study
EibD-induced	BL21 ∆ABCF	pBAD/HisA	Induction for determining surface expression	This study*
EibG-induced	BL21 ∆ABCF	pBAD/HisA	Induction for determining surface expression	This study*
EibD-induced_2	TOP10	pBAD/HisA	Induction for determining surface expression	This study*
EibG-induced_2	TOP10	pBAD/HisA	Induction for determining surface expression	This study*
EibG-SpyTag	*E. coli* TOP10	pASK-IBA2con_*eibG*-SpyTag	EibG variant with a 5’ Spy-Tag	This study
EibD-SpyTag	*E. coli* TOP10	pASK-IBA2con_*eibD*-SpyTag	EibD variant with a 5’ Spy-Tag	This study

Strain names, the host with the obtained plasmids, as well as a short description and reference are listed. Deleted amino acids are shown in square brackets. Control strain was obtained from Invitrogen, thermo fisher Scientific, Waltham, MA, USA. *The pBAD-EibD/EibG were constructed based on pBAD/HisA from Invitrogen.

### Cloning and mutagenesis

For inducible production of EibG and EibD, the coding sequences for the proteins were amplified from pGEMEBG^27^ and pETDuetS-EibD^53^, respectively. These were cloned into the arabinose-inducible vector pBAD/HisA (Invitrogen). To produce a plasmid giving low-level constitutive expression, we replaced the *tet* promoter of pASK-IBA2C (IBA Lifesciences GmbH, Goettingen, Germany) with the constitutive promoter J23105 (https://parts.igem.org/Part:BBa_J23105) by site-directed mutagenesis^54^. *eibD* (with its restored original signal peptide) and *eibG* were subcloned into this vector, as were *eibA* (with an EibD signal peptide, from pETDues-EibA^53^ and YadA (from *Yersinia enterocolitica* O:3, amplified from genomic DNA of strain 6471/76^55^. For fluorescent labelling, we used either mNeonGreen or mCherry, the coding sequences for which were inserted into the plasmid pACYC184^56^. The SpyTag^38^ sequence was introduced using mutagenesis primers to insert the tag into the pASK-IBA2C-eibG and pASK-IBA2C-eibD vectors, respectively, at the 5′ end of the genes to produce N-terminally tagged proteins. All cloning procedures were performed with Gibson assembly^57^ using the 2 x HiFi Master Mix (New England Biolabs, Ipswich, UK) and VeriFi™ Mix Red (PCR Biosystems Ltd., London, UK) for amplification of fragments or Q5 polymerase (New England Biolabs, Ipswich, UK) for site-direct mutagenesis. All primers are listed (Supplementary Table 1). Domain deletions were performed based on a previously published protocol^54^. The annotation of domains was performed based on AlphaFold^58^ (Fig. 2 and Supplementary Fig. 3b). Chemically competent *E. coli* TOP10 cells^59^ were transformed with the respective cloning products. Clones were validated by colony polymerase chain reaction using PCRBIO Taq Mix Red (PCR Biosystems Ltd) and Sanger sequencing (Source Bioscience Genomics, Cambridge, UK).

### Bright field and epifluorescence microscopy

Chain formation was evaluated using bright field microscopy. Overnight cultures were diluted (1:50) in 10 mL of fresh medium and incubated at 37 °C with shaking at 30 rpm for at least 5 h. After incubation, 1 mL of the culture was gently transferred to a 2 mL microcentrifuge tube using a 1000 µL pipette tip with the tip cut to minimize shear stress on the bacteria. The sample was allowed to sediment for 30 min at room temperature. During the sedimentation period, microscopy slides were prepared using 1 mL of 1% agarose in phosphate buffered saline (PBS) on each slide, allowing it to solidify. Following sedimentation, the bacteria were resuspended by gently pipetting up and down three times with the cut tip. A small drop of the resuspended bacteria was transferred to a prepared microscopy slide. Microscopy was performed using a Nikon Eclipse epifluorescence microscope (Nikon, Amstelveen, The Netherlands) at 1000x magnification with immersion oil. Potential formation of mixed chains was assessed by using TOP10 cells either expressing EibG and mNeonGreen or EibD and mCherry, or TAA-expressing cells (non-fluorescent) with TOP10 cells expressing mNeonGreen. mNeonGreen and mCherry were introduce on a pACYC184-based plasmid, either with chloramphenicol resistance or tetracycline resistance. The samples were prepared with minor modifications. Individual overnight cultures of the described strains (with chloramphenicol or chloramphenicol and tetracycline for mNeonGreen/EibG and mCherry/EibD strains) were equally diluted in fresh medium (OD_600_ 0.05) and incubated at 37 °C with shaking at 30 rpm for at least 5 h. Bright field and epifluorescence mode were used simultaneously to identify (i) Eib and NeonGreen (Eib-negative) expressing cells respectively or (ii) EibG and mNeonGreen or EibD and mCherry expressing cells. Overlay of images was ensured using microscopic software (NIS-Elements, Nikon v.5.11.01).

### Quantification of clump and chain formation

For quantification of clump and chain formation compared to individual cells we used the Fiji software^60^. For that, microscopic images were exported as tagged image files and imported into Fiji. Ten semi-randomly taken microscopic images were used from four biological replicates each. If multiple color channels exist, they were reduced to one and background removal was performed. The threshold was adjusted and the particles were analyzed in default mode to identify the cells. Shape descriptions were measured as the read out analyzing the individual particles (Size: 0-Infinity; Circularity: 0.00–1.00).

### Expression and purification of SpyCatcher-sfGFP and SpyCatcher-EQ-sfGFP

SpyCatcher proteins were produced as described before with minor modifications^38^. In brief, *E. coli* BL21Gold(DE3) cells harboring either pET22-SpyCatcher-sfGFP or pET22-SpyCatcherEQ-sfGFP plasmids were inoculated separately into 5 mL AIM supplemented with 100 µg/mL ampicillin in the morning and grown at 37 °C for several hours. The cultures were then transferred into 1 L of AIM containing 100 µg/mL ampicillin and antifoam for incubation overnight at 37 °C in a LEX10 bioreactor (Epiphyte3, Toronto, Ontario, Canada). The following day, cells were harvested by centrifugation (15 min at 5000 x *g*) and resuspended in 30 mL PBS, then stored at − 20 °C until purification. Frozen cell suspensions were thawed and treated with 0.1 mg/mL of lysozyme, a pinch of DNase I, 1 mM MgCl_2_, and 1 mM MnCl₂. Bacterial cells were lysed using a probe sonicator for six 30-second cycles. The lysate was clarified by centrifugation at 30,000 x *g* for 60 min. The supernatant was applied to a Ni-NTA affinity column connected to an FPLC unit. Proteins were eluted using an imidazole gradient (10 mM – 500 mM). Fractions were collected, and 40 µL aliquots from each were prepared for sodium dodecyl sulfate–polyacrylamide gel electrophoresis (see Supplementary methods).

### Evaluation of protein localization using spycatcher and confocal microscopy

The EibG-SpyTag and EibG-SpyTag expressing-strains were grown overnight, diluted in the morning and incubated at 37 °C with shaking at 50 rpm. After 4 h of growth, SpyCatcher-sfGFP or SpyCatcher-sfGFP-EQ was added to the culture to a final concentration of 0.02 mg/mL. The culture was gently swirled to ensure proper mixing and then incubated for an additional 1.5 h at 37 °C with shaking reduced to 30 rpm. After the incubation period, the bacterial clumps were allowed to settle for a few minutes without shaking. A 400 µL sample from the bottom of the tube was transferred to a 2 mL tube and carefully resuspended, using a pipette with the tip cut off. To wash the cells, 400 µL PBS was added and the mixture was centrifuged at 1000 x *g* for 3 min. The supernatant was discarded, and the bacterial pellet was gently resuspended in 800 µL PBS with minimal pipetting, followed by gentle swirling and flicking of the tube to remove the bacteria from the bottom. The washing step was repeated. Afterwards, the bacterial pellet was gently resuspended in 200 µL of PBS and a 6 µL aliquot of the resuspended sample was transferred to an agarose pad using a pipette with a cut-end tip for imaging using Nikon AXR with NSPARC. To analyze the fluorescence intensity at the bacterial cell-to-cell contact points, images were processed using Fiji software^60^. A single bacterial chain was first selected using the selection tool. This selection was duplicated to preserve the original image and facilitate further analysis. A maximum intensity projection of the Z-stack was created. The fluorescence image was subsequently split into individual color channels. The area corresponding to cell-to-cell contact was identified within the bright field image using the freehand selection tool. The selection was transferred to the fluorescence channel. The selected region of interest (ROI) was added to the ROI manager and mean fluorescence intensity within this ROI was measured. This was repeated for all cell-to-cell contacts within the chain and then for the whole chain as well. The data were then exported. To evaluate differences in fluorescence intensity, statistical analysis was performed using an R-script to generate plots and conduct a paired t-test. This test compared the fluorescence signal across the entire bacterial chain with the mean fluorescence intensity at the cell-to-cell contact points to determine if significant differences existed.

### Sedimentation

Overnight cultures (10 mL) were inoculated from single cultures and grown for 16 h overnight at 37 °C shaking at 30 rpm. The following day, the cultures were carefully inverted to ensure uniform mixing and transferred into long glass tubes. The OD_600_ was measured every 15–30 min by taking 200 µL from the top pf the cultures using the plate reader (SPECTROstar Nano; BMG Labtech, Ortenberg, Germany). The cultures were incubated at 37 °C statically in-between. Reduction in OD_600_ relative to the initial OD was measured as degree of sedimentation.

### Crystal Violet assay

For analyzing biofilm formation, the OD_600_ of overnight cultures was adjusted to 0.2 and 100 µL of OD-adjusted culture was inoculated into four wells of a Nunc™ MicroWell™ 96-Well-mikrotiter plate (ThermoFisher Scientific, Waltham, MA, USA). As negative control, the respective medium alone was included. Plates were sealed with PARAFILM^®^, and incubated statically at 37 °C for 48 h. Afterwards, the culture was carefully aspirated and the adhered biofilm was washed three times with 200 µL of PBS to remove non-adherent cells. The plates were then incubated at 60 °C for at least 60 min to fix the biofilm. The adhered biofilm was stained by adding 150 µL of 1% (w/v) crystal violet (Sigma Aldrich; Merck, Darmstadt, Germany) for 20 min. The plates were rinsed three times with distilled water and left inverted to dry. To solubilize the stained biofilm, 150 µL of 33% (v/v) glacial acetic acid (Scientific Laboratory Supplies, Nottingham, UK) was added per well. After complete solubilization, the solution was transferred to a new plate. The OD at 540 nm (OD_540_) was measured (SPECTROstar Nano; BMG Labtech).

### Software and statistics

For all cloning approaches, we used SnapGene Viewer (GSL Biotech LLC v7.1.2.0) in combination with ApE-A plasmid Editor (v3.1.6) and Oligo Calculator (v3.27)^61^. Evaluation of microscopy was done with Nikon NIS (v5.11) together with Fiji (ImageJ Version 2.35). NIS Element (v6.10.01). Data evaluation and visualization was performed using GraphPad Prism (v10.2.1) and RStudio (v2023.06.2 + 561). Paired t-test or One-way ANOVA with Dunnett’s post-hoc test was used for multiple comparisons when applicable. We used large language models, such as ChatGPT (GPT-4), to assist in revising portions of the manuscript. All AI-generated content was thoroughly reviewed, edited, and approved by the authors to ensure its accuracy and integrity.

## Supplementary Information

Below is the link to the electronic supplementary material.

Supplementary Material 1

## Data Availability

Raw data microscopic images and metadata are stored at FigShare (https://doi.org/10.6084/m9.figshare.29166725.v1). Gene and plasmid sequences are publically available and can be found at NCBI ( https://www.ncbi.nlm.nih.gov ) as: *eibA* (AF151091.1), *eibD* (AAF63040.1), *eibG* (ADJ17717.1), *yadA* (WP_032488477.1) or pASK-IBA2C (2-1321-000; IBA Lifesciences GmbH, Goettingen, Germany; https://www.iba-lifesciences.com/de/pask-iba2c-vector/2-1321-000). The tet promoter of pASK-IBA2C was replaced with the constitutive promoter J23105 (https://parts.igem.org/Part: BBa_J23105) by site-directed mutagenesis.

## Abbreviations

AIM: Auto-induction medium
CLAP: Chain-like adherence pattern
Eib: *E. coli* immunoglobulin-binding proteins
GFP: Green fluorescent protein
Ig: Immunoglobulin
LPBR: Left-handed parallel β-roll
NTD: N-terminal domain
OD_540_: Optical density at 540 nm
OD_600_: Optical density at 600 nm
PBS: Phosphate buffered saline
ROI: Region of interest
SDS-PAGE: Sodium dodecyl sulfate–polyacrylamide gel electrophoresis
sfGFP: Superfolder green fluorescence protein
STEC: Shiga toxin-producing *E. coli*
TAAs: Trimeric autotransporter adhesins
