# Supplementary material for: Chain formation mediated by Escherichia coli immunoglobulin-binding proteins EibD and EibG depends on expression levels and localization of proteins
Source: Sci Rep. 2025 Oct 9;15:35244. doi: 10.1038/s41598-025-22967-3 (PMC12511318; doi:10.1038/s41598-025-22967-3)
Supplement: Supplementary file 1 — Supplementary Material 1 [file 41598_2025_22967_MOESM1_ESM.docx]

## Supplement

### Supplementary methods

### Outer membrane extraction

Outer membrane extraction for validation of protein expression was performed based on Carlone 1986 et al. [1] with slight modifications. In brief, cultures (50 mL) were incubated overnight. The following day, an amount of cells corresponding to 50 mL of an optical density at 600 nm (OD_600_) equaling 1 were harvested (10 min. 5,000 *x* g). Pellets were resuspended in 1 mL cold lysis buffer (10 µg/ml DNase I, 100 µg/ml lysozyme, 1 mM MgCl2 in 10 mM HEPES pH 7.5). Mechanical cell disruption was ensured by using a cell homogenizer (Precellys 24 Touch; Bertin Technologies SAS, Montigny-le-Bretonneux, France) for 2x 40 sec with cooling in-between. Cell debris was pelleted (2 min, 10,000 *x* g) and supernatant transferred into a fresh tube to pellet membranes (30 min, 16,000  *x* g, 4°C). Pellets were resuspended in 200 µL 0.1 M HEPES pH 7.5. An equal volume of 2% *N*-lauroyl sarcosine in 10 mM HEPES and samples were incubated (30 min, 300 rpm, 22°C) to solubilize the inner membranes. Afterwards, outer membranes were pelleted (30 min, 16,000  *x* g, 4°C). Pellets were washed with 0.5 ml 10 mM HEPES pH 7.5 and centrifugation was repeated for 10 min. The pellets were then resuspended in 40 µL 10 mM HEPES pH 7.5, 10 µL sodium dodecyl sulfate–polyacrylamide gel electrophoresis (SDS-PAGE) sample buffer was added, and samples were stored at -20°C until further use.

### SDS-PAGE

Samples were prepared by heating at 50°C for 10 min to not disrupt the TAA trimers. Then, the gel (Novex 4-20% Tris-glycine gel; Thermo Fisher Scientific, Waltham, MA, USA) was loaded with 15 µL of each sample and 2.5 µL of the size standard (Pageruler Prestained Protein Ladder; Thermo Fisher Scientific). Electrophoresis was performed for 1 h at 25 mA per gel. A colloidal comassie brilliant blue stain (PageBlue, Thermo Scientific) was used for visualization of protein bands.

### Macrocolony formation

Assessing biofilm phenotype was performed by observeing macrocolony formation of long-term agar consisting of LB-Lennox (Carl Roth, Germany) and Span agar (H. Carroux, Germany) A sterile Congo red-Coomassie-Brilliant-Blue G250 solution (50 mg/L Congo red, 25 mg/L Coomassie-Brilliant-Blue G250 solved in 20 mL 87.5% ethanol) was added. Bacterial cultures were inoculated with a single colony grown on LB-agar and incubated overnight at 37 °C shaking (180 rpm). From each culture 5 µL were spotted onto a freshly prepared agar plate. Plates were sealed with PARAFILM® and incubated at 28 °C for five days.

### Supplementary tables

Supplementary table 1: **List of primers used in this study**. The orimer name, sequence and the application are given.

| Name | Sequence (5‘ 🡪 3‘) | Application |
| --- | --- | --- |
| EibD/A_F | TAACGAGGGCAAAAAATGAAATACCTGCTGCCGACC | Amplification of *eibD* and *eibA* |
| EibD/A_R | CACAGGTCAAGCTTATTAAAACTCGAAGTTCACACCAACGT | Amplification of *eibD* and *eibA* |
| EibG_F | TAACGAGGGCAAAAAATGAGTAAAAAGTTTACAATGACACTTC | Amplification of *eibG* |
| EibG_R | CACAGGTCAAGCTTATTAAAACTCGAAGTTAACGCCCAT | Amplification of *eibG* |
| YadA_F | TAACGAGGGCAAAAAATGATGACTAAAGATTTTAAGATCAGTGTC | Amplification of *yadA* |
| YadA_R | GTGGCTCCAAGCGCTCTATTACCACTCGATATTAAATGATGCAT | Amplification of *yadA* |
| IBAseq_F | GGTACTATGCTAGCCCCT | Sequencing |
| IBAseq_R | CGTTTACCGCTACTGCG | Sequencing |
| EibGmutR | AGCTAAAACACCTGAGCTGATC | Revers primer for domain deletions and exchanges (amplification of *eibG*-plasmid) |
| EibG∆NtermF | CTCAGGTGTTTTAGCTGTTCAGGCTAGTGCGGCG | Deletion of N-terminal domain |
| EibGBBf | GGTCTTTTCCAGCCGTACAG | Exchange up to and including the LHCC domain; *eibG* template |
| EibG∆HeadF | CTCAGGTGTTTTAGCTCGTCAGGTTGACCGTGTAAAA | Deletion up to and including the neck domain |
| EibG∆RHccF | CTCAGGTGTTTTAGCTCGTCAGGTTGACCGTGTAAAA | Deletion up to and including the saddle domain |
| EibG-EibDNtermR | CGCCGCACTAGCCTGAACTTCGACGCTGAAACCTGTAATTTC | Exchange of N-terminal domain; *eibD* template |
| EibG-EibDNtermF | AGCTCAGGTGTTTTAGCTCAGAATGGTACATATTCAGTGTTG | Forward primer for exchange of domains; *eibD* template |
| EibG-EibDstalkR | GTACGGCTGGAAAAGACCTGTCAGGGCCGCAGACTG | Exchange up to and including the LHCC domain; *eibD* template |
| EibGRHccF | CGTCAGGTTGACCGTGTAAAA | Exchange up to and including the neck domain; *eibG* template |
| EibG-EibDneckR | TACACGGTCAACCTGACGAACAGTTGCCGCATCACTGTT | Exchange up to and including the neck domain; *eibD* template |
| EibGLPBRf | GTTCAGGCTAGTGCGGCG | Exchange of N-terminal domain; *eibG* template |
| EibG∆LPBRf | GAAATATTAAAACAATCGCGTCAGGTTGACCGTGTAAAA | Deletion of LPBR domain |
| EibGNtermR | CGATTGTTTTAATATTTCAGGATTAATAAT | Deletion of LPBR domain |
| EibG∆Pass | CTCAGGTGTTTTAGCTCACGAAGAGATGAAGCGTGC | Deletion of the passenger |
| pBAD-EibDf2 | CAGGAGGAATTAACCATGAAATACCTGCTGCCGACCG | Amplification of *eibD* for cloning into pBAD |
| pBAD-EibDr2 | GCCAAAACAGCCAAGCTTTTAAAACTCGAAGTTCACACCAACG | Amplification of *eibD* for cloning into pBAD |
| pBAD-EibGf | CAGGAGGAATTAACCATGAGTAAAAAGTTTACAATGACACTT | Amplification of *eibG* for cloning into pBAD |
| pBAD-EibGr | GCCAAAACAGCCAAGCTTTTAAAACTCGAAGTTAACGCCCAT | Amplification of *eibG* for cloning into pBAD |
| pBADRecF | AAGCTTGGCTGTTTTGGCGGA | Amplification of pBAD for cloning *eib*s |
| pBADRecR | CATGGTTAATTCCTCCTGTTAGC | Amplification of pBAD for cloning *eib*s |
| SpyTag-EibGf | ATGGTGGATGCATATAAACCGACCAAACAGGAGTTACCTCCTATCAAAG | Insertion of SpyTag |
| SpyTag-EibGr | ATATGCATCCACCATAACAATATGGGCAGCTAAAACACCTGAGCTGATC | Insertion of SpyTag |
| SpyTag-EibDf | ATGGTGGATGCATATAAACCGACCAAACAGAATGGTACATATTCAGTGTTG | Insertion of SpyTag |
| SpyTag-EibDr | ATATGCATCCACCATAACAATATGGGCCGCACTCACTCCACCACT | Insertion of SpyTag |

### Supplementary figures


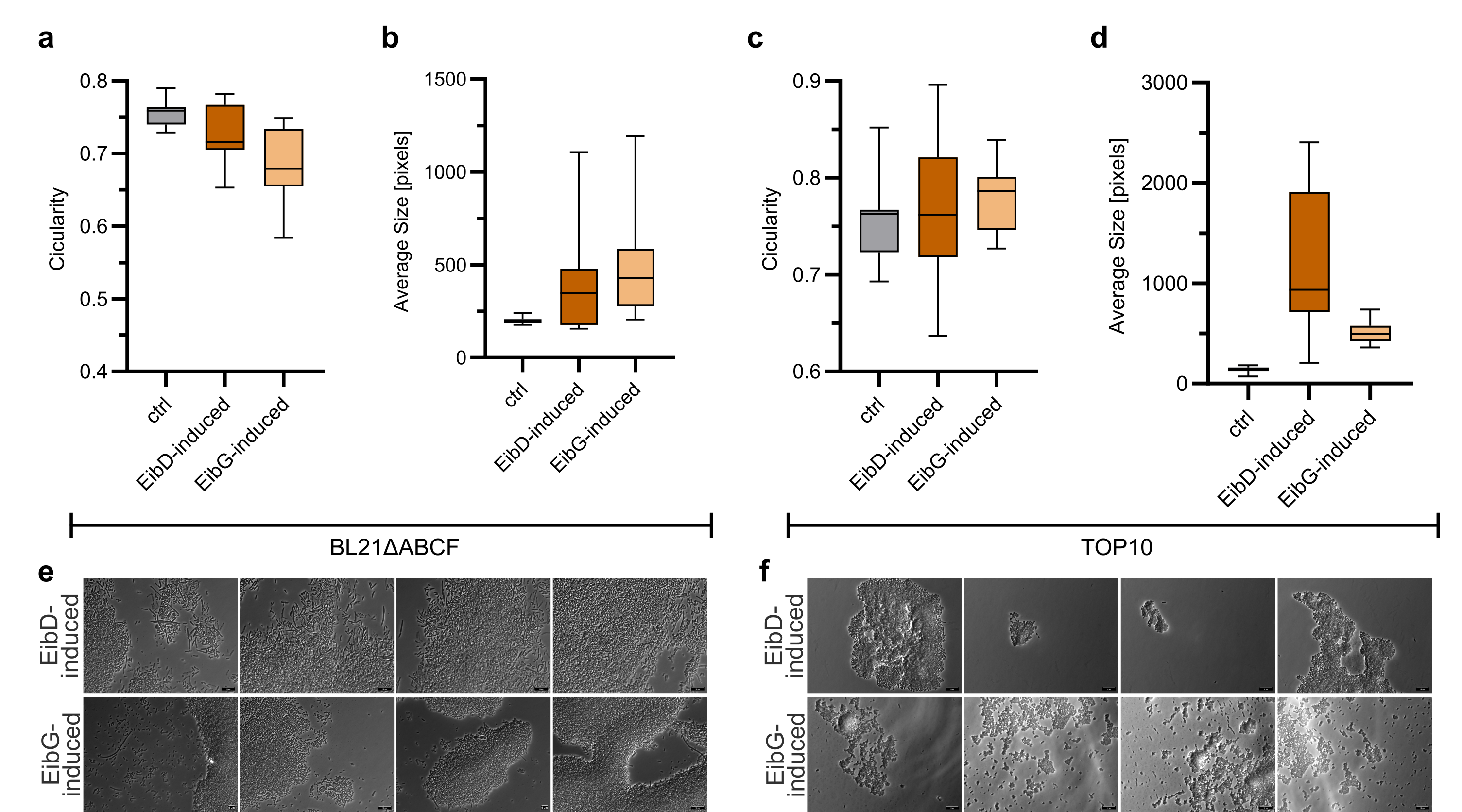


Supplementary figure 1: **Bright field microcopy and quantification of clump formation.** (**a**+**b**+**e**) BL21ΔABCF cells or (**c**+**d**+**f**) TOP10 cells transformed with the indicated constructs were cultivated in auto-induction medium[2] containing 0.2% l‑arabinose for induction of EibD or EibG expression respectively and afterwards assed for auto-aggregation by bright field microcopy. The scale bar in the bottom right corner indicates 10 µm. (**a**-**c**) Eleven semi-randomly taken pictures were evaluated to obtain their (**a**+**c**) circularity and (**b**+**d**) size using Fiji (ImageJ). Boxplots show the median, interquartile range (25th to 75th percentile), and whiskers representing the minimum and maximum values.


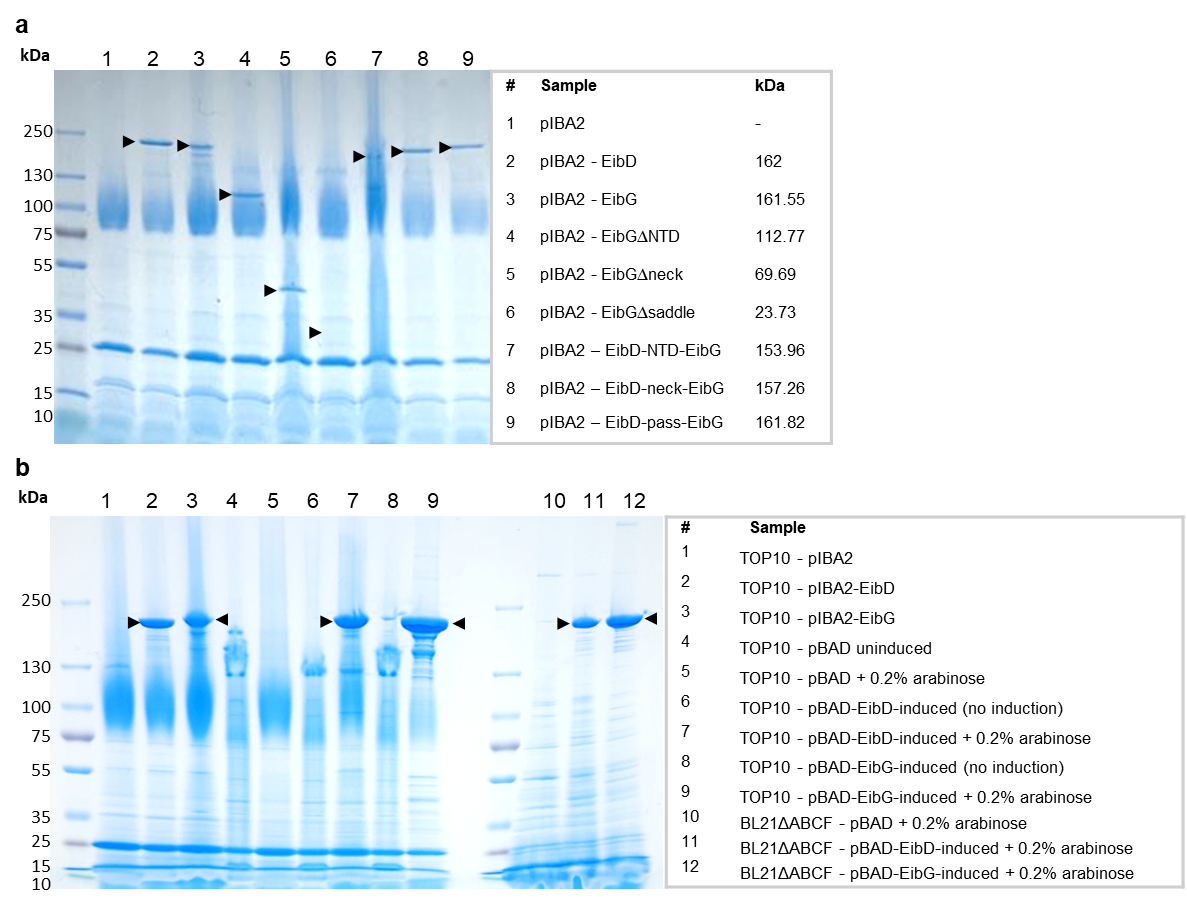


Supplementary figure 2: **SDS-PAGE of outer membrane preparations from TOP10 or BL21ΔABCF cells expressing different Eib variants.** Strains (TOP10 or BL21ΔABCF) transformed with indicated plasmids (pIBA2 or pBAD) were cultivated in LB overnight and expression was induced with 0.2% arabinose where indicated. Cells corresponding to 10 mL of OD_600nm_=1 were collected for outer membrane preparation. An SDS-Gel (4-12%) was loaded with 15 µL samples each of (**a**) all indicated pIBA2 Eib construct in TOP10 cells and (**b**) TOP10 or BL21 ΔABCF with constitutive expression using pIBA2 plasmids or induced expression using pBAD plasmids.


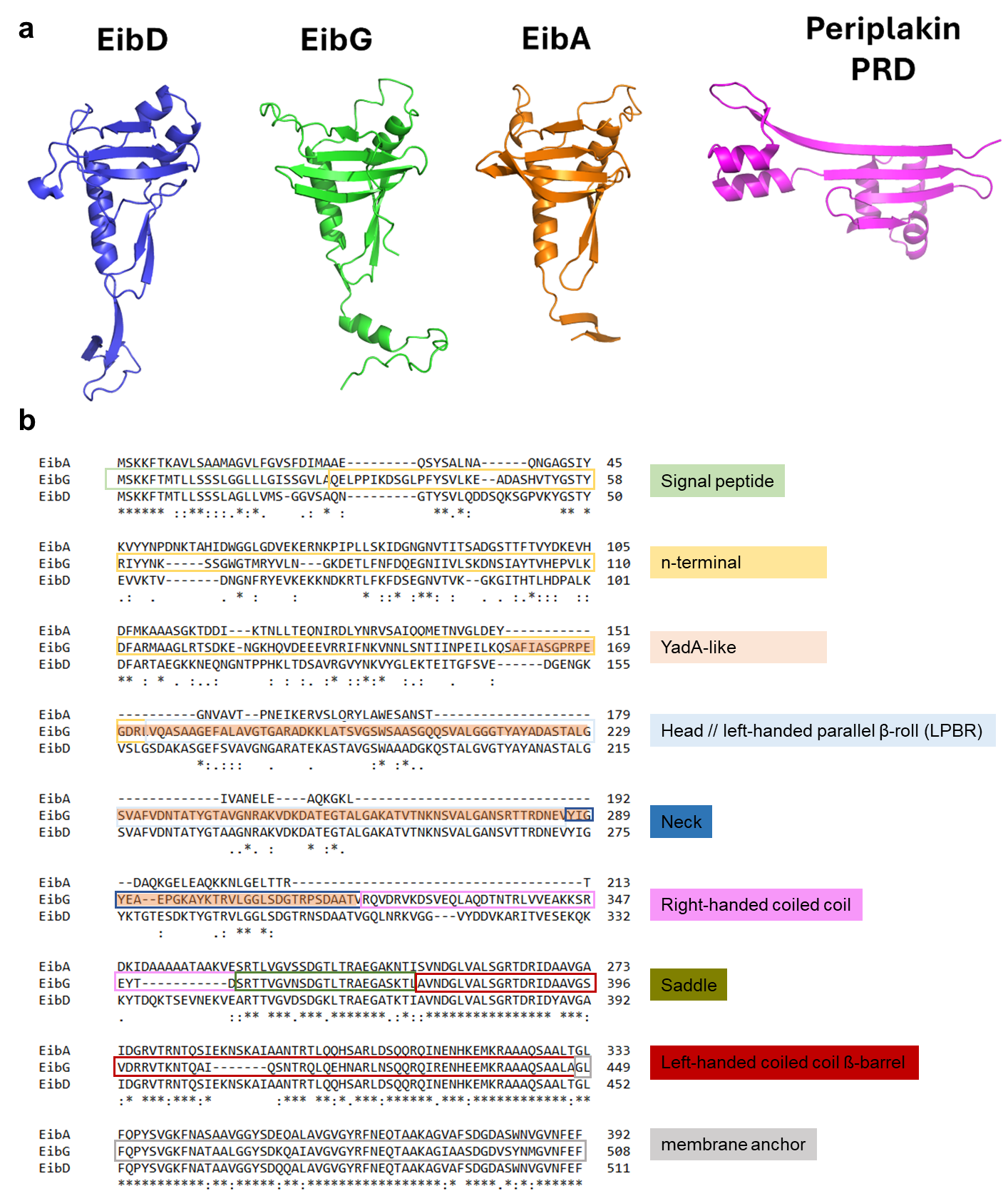


Supplementary figure 3: **Annotation of analyzed domains.** (**a**) Structures of the Eib NTDs: A monomer of the NTDs is shown, with EibD in blue, EibG in green and EibA in orange. They share a similar triangular core architecture, but differ in the loops. The figures include the connector to the next domain/region at the C-terminus, which is different for the three proteins: EibA and EibD connect via a β-hairpin, which has a longer loop in EibD, whereas EibG connects through a short α-helix leading into a β‑hairpin stacking with the LPBR domain. A DALI search[3] identified the plectin repeat domain (PRD) of periplakin (PDB ID: 4Q28) as having stuctural similarity with the Eib NTDs (Z score 3.9). This structure is shown in magenta. (**c**) The different structural and functional domains of the Eib proteins are highlighted in the indicated color. Alignment of the different Eib proteins EibA, EibD, and EibG was performed with Clustal Omega Multiple Sequence Alignment (MSA).


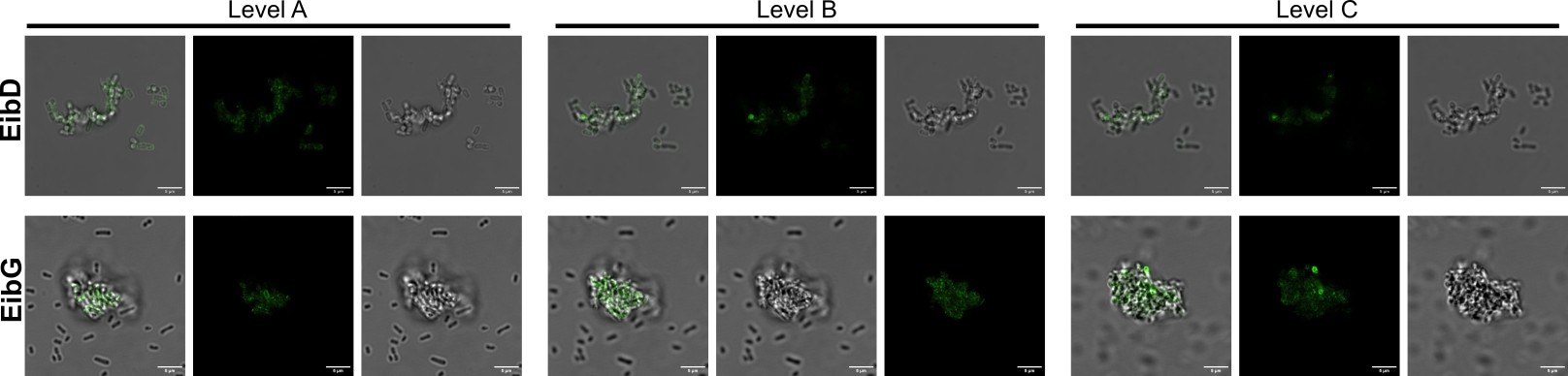


Supplementary figure 4: **Z-stack images of clumps formed by cells expressing EibD-SpyTag or EibG-SpyTag.** *E. coli* cells expressing either EibD-SpyTag or EibG-SpyTag were incubated with purified SpyCatcher protein to detect surface-exposed SpyTag. Samples were prepared for confocal microscopy to analyze the spatial distribution of the fluorescent signal. Three representative Z-planes are shown for each condition, illustrating the localization of fluorescence within bacterial clumps. The scale bar in the bottom right corner indicates 5 µm.

**
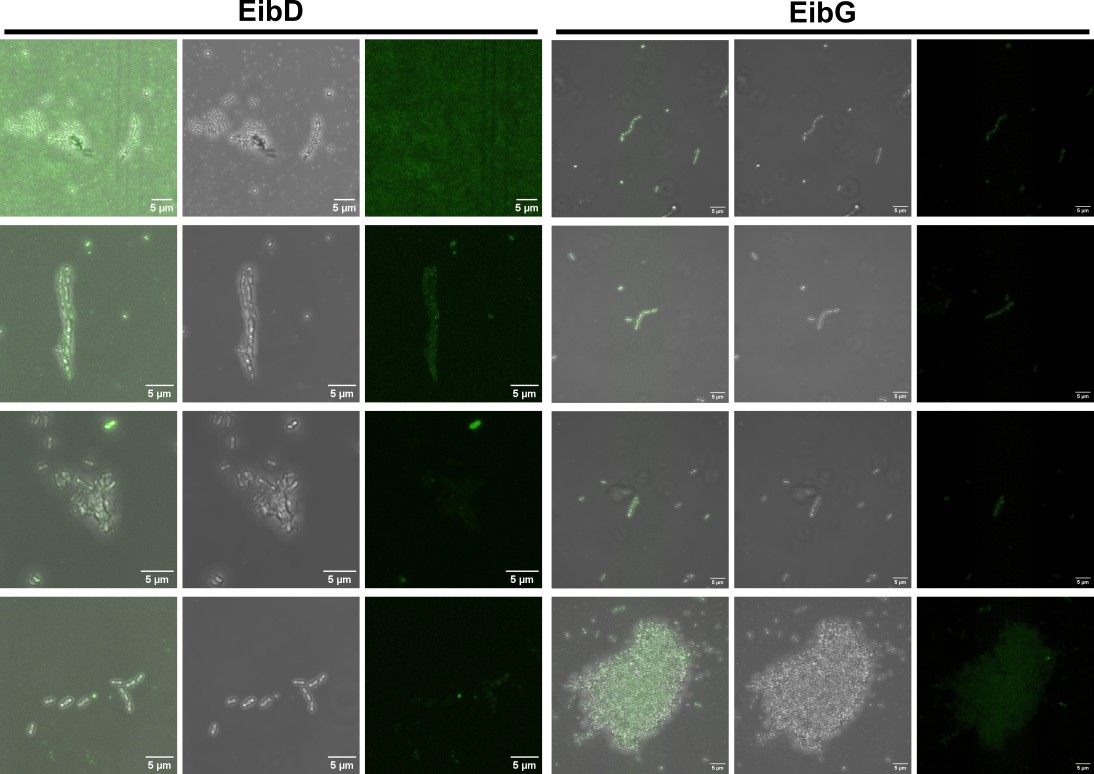
**

Supplementary figure 5: **Cells expressing EibD-SpyTag or EibG-SpyTag stained with SpyCatcher-EQ variant.** Regardless of the Eib protein, the staining of individual cells, chains, or clumps with the SpyCatcher-EQ resulted in diffusive non-specific signals. The scale bar in the bottom right corner indicates 5 µm.


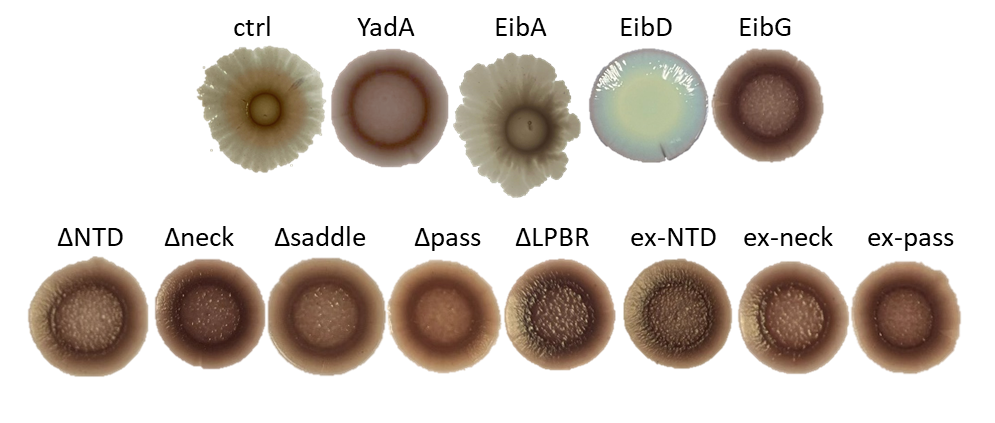


Supplementary figure 6: **Marcocolony formation on long-term agar plates.** Liquid cultures of the indicated stains (ex = exchange between EibD and EibG) were spotted onto long-term agar plates containing Congo red and Coomassie-Brilliant blue for staining of the curli biofilm structures. Plates were sealed with PARAFILM® and incubated at 28 °C for five days.
